# Collecting Evidence to Inform Salt Reduction Policies in Argentina: Identifying Sources of Sodium Intake in Adults from a Population-Based Sample

**DOI:** 10.3390/nu9090964

**Published:** 2017-08-31

**Authors:** Natalia Elorriaga, Laura Gutierrez, Iris B. Romero, Daniela L. Moyano, Rosana Poggio, Matías Calandrelli, Nora Mores, Adolfo Rubinstein, Vilma Irazola

**Affiliations:** 1 Centro de Excelencia en Salud Cardiovascular para el Cono Sur (CESCAS), C1414CPV Ciudad Autónoma de Buenos Aires, Argentina; lgutierrez@iecs.org.ar (L.G.); dmoyano@iecs.org.ar (D.L.M.); rpoggio@iecs.org.ar (R.P.); virazola@iecs.org.ar (V.I.); 2 Escuela de Nutrición, Universidad de Buenos Aires, C1122AAD Ciudad Autónoma de Buenos Aires, Argentina; irisbromero@yahoo.com.ar; 3 Sanatorio San Carlos, Pcia de Río Negro, 8400 Bariloche, Argentina; matiascalandrelli@yahoo.com.ar; 4 Municipalidad de Marcos Paz, Pcia de Buenos Aires, 1727 Marcos Paz, Argentina; oliveramores@gmail.com; 5 Ministerio de Salud de la Nación, C1073ABA Ciudad Autónoma de Buenos Aires, Argentina; adolfo.rubinstein@gmail.com

**Keywords:** salt, sodium intake, food sources, processed foods, Argentina, adults, food frequency questionnaire, food policy

## Abstract

The maximum content of sodium in selected processed foods (PF) in Argentina was limited by a law enacted in 2013. Data about intake of these and other foods are necessary for policy planning, implementation, evaluation, and monitoring. We examined data from the CESCAS I population-based cohort study to assess the main dietary sources among PF and frequency of discretionary salt use by sex, age, and education attainment, before full implementation of the regulations in 2015. We used a validated 34-item FFQ (Food Frequency Questionnaire) to assess PF intake and discretional salt use. Among 2127 adults in two Argentinean cities, aged 35–76 years, mean salt intake from selected PFs was 4.7 g/day, higher among male and low education subgroups. Categories of foods with regulated maximum limits provided near half of the sodium intake from PFs. Use of salt (always/often) at the table and during cooking was reported by 9% and 73% of the population, respectively, with higher proportions among young people. Reducing salt consumption to the target of 5 g/day may require adjustments to the current regulation (reducing targets, including other food categories), as well as reinforcing strategies such as education campaigns, labeling, and voluntary agreement with bakeries.

## 1. Introduction

Noncommunicable diseases (NCDs) are the main contributor to mortality and morbidity globally [1,2] and interventions to reduce the burden of NCDs are highly cost-effective [3]. Elevated sodium intake has been associated with a number of NCDs (including hypertension, cardiovascular disease, and stroke), and decreasing sodium intake may reduce blood pressure and the risk of associated NCDs [4]. In Argentina, cardiovascular disease (CVD) is the first cause of death in the general population [5] and 37% of all cardiovascular deaths in Argentina are attributable to hypertension [6]. Hypertension is more frequent among those with the lowest educational level and lowest income subgroups [7]. Although no studies have measured total sodium consumption in a population-based sample of Argentina, consensus among experts suggests that current sodium intake is at least double of the WHO 2000 mg/day recommendation [8].

Population-based interventions to reduce sodium intake are being successfully implemented in various countries worldwide, and have the potential to reduce the prevalence of high blood pressure and the burden of cardiovascular diseases [9]. The Argentinean government has shown leadership in developing strategies to reduce sodium consumption. In 2009, the national program called Menos Sal Más Vida (“Less Salt More Life”) was launched, promoting the reduction of salt consumption by the Argentinean population [10]. It has included actions like a voluntary agreement with the Argentinean Federation of Bakeries (FAIPA, by its Spanish acronym) to reduce salt levels in breads [8]. In 2011, the initiative was consolidated through the signing of agreements for Voluntary and Progressive Reduction of the Sodium content of the Processed Foods celebrated between the Ministry of Health (MoH), the Ministry of Agriculture, Livestock and Fisheries and large food industries of the country [4]. The initiative required food companies to reduce 5% to 15% of the sodium content of four groups of food: (1) processed meats; (2) cheese and dairy products; (3) soups and dressings; and (4) cereals, cookies, pizza, and pasta (farinaceous). In 2013, National Act 26905 [11] was passed, establishing different lines of action in order to strengthen public health policies to promote the reduction of the consumption of sodium. One of the strategies was the definition of mandatory maximum levels of sodium in selected processed foods included in the former voluntary agreement, with the MoH being the implementation authority to set new progressive and gradual reduction targets and to include new food categories. In 2017, according to the Joint Resolution 1-E/2017 [12], these maximum limits were included in the Argentinean Food Code (CAA, by its Spanish acronym).

A key component of any sodium reduction intervention must be monitoring sodium consumption at the population level [9]. It should provide essential information to policymakers and all interested stakeholders on the population levels of sodium consumption; the main dietary sources of sodium; the goals and objectives to be reached and the progress, and limitations and results of the implementation of the intervention [2]. As was previously mentioned, measurements of total sodium intake through 24 h urinary excretion at the national level in Argentina have not been undertaken yet, but using the spot urines from a pilot study conducted in the province of La Pampa, a mean sodium consumption of 4832 mg/day for men, 3983 mg/day for women, and 4407 mg/day on average, was estimated, equivalent to 12.1, 10, and 11 grams/day of salt, respectively [13]. Although urinary sodium excretion can indicate the magnitude of the problem within a population, neither the sources of sodium intakes nor the means to reduce it can be identified through this measure. A complete determination of sodium sources involves assessment of several separate elements including dietary intake, sodium content of food consumed, and discretionary salt use in cooking or at table [9]. Frequency of discretionary salt use at the table has been assessed at the three waves of the National Risk Factor Surveys (NRFS) [7,14,15], showing that population that have reported adding always or almost always salt at the table represented 23.1%, 25.3%, and 17.3% in 2005, 2009, and 2013. Discretionary salt use in cooking has been less studied and, according to qualitative studies, it may be an important source of sodium in Latin America [16,17]. Updated information on diet sources of sodium from studies with individual data at the national level is scarce, but based on data from National Health and Nutrition Survey (ENNyS, by its Spanish acronym) conducted in 2004–2005 among children and women less than 50 years, the MoH estimated that between 65% and 70% of dietary sodium intakes come from processed foods [8]. A recent work analyzing sodium content in processed foods based on nutritional panels has indicated that in 2014, before the national law had entered into force, most of the regulated products were below or at the upper-levels defined by the regulation [18]. However, full interpretation of the achieved reduction of sodium content in these foods required dietary intake data of these food products. Henceforth, some knowledge gaps should be filled to effectively guide policy development and monitoring; mainly: (a) which are the main food products that are currently providing most of the sodium at the population level; (b) how much sodium are they contributing? (c) how often is the consumption of low-salt food alternatives? (d) do the main sources differ across subgroups of age, sex, and level of education? (e) are the implemented strategies covering the main sources of sodium at different population groups? Although the national level is the target for a national policy, and a second wave of the ENNyS is planned to be conducted in the short-term, before those results are ready, analysis of dietary population-based information at sub-national levels will be useful to feed the policy implementation process by supporting interventions tending to achieve the goal of consuming less than 5 g of salt per day.

Thus, the purpose of this study is to contribute to the implementation, monitoring, and evaluation of salt reduction policies and the design of new courses of action by providing policy makers and stakeholders with valuable information about the main diet sources of sodium among adults in Argentina and their variation across subgroups of sex, age, and level of education. With this aim, we used Argentinean data from a Southern Cone multicentric population-based cohort study, to examine estimated sodium intakes from selected food products, the consumption of main available low-salt alternatives, the frequency of discretionary salt use in cooking and at the table among adults in 2014, before the full implementation of the national law.

## 2. Materials and Methods 

### 2.1. Design and Population

We conducted a cross-sectional study in a random subset of participants of the CESCAS I (Centro de Excelencia en Salud Cardiovascular para América del Sur) Study [19]. Details of the study design and sampling methods of the CESCAS I study have been published elsewhere [20]. Briefly, a random multiple-stage stratified sampling was used to select representative samples of the general population, aged 35–74 years old from four cities in Latin America: Bariloche and Marcos Paz in Argentina, Temuco in Chile and Canelones in Uruguay. The sampling design included four stages: In the first stage, census enumeration areas were randomly selected in each location using probability proportional to size, with stratification by socioeconomic level of the enumeration area. In the second stage, blocks per census enumeration area were also randomly selected. In the third stage, households from each block were selected through systematic random sampling methods. In the fourth stage, one household member between 35–74 years old was selected for the study. 

In the present study, we assessed the frequency of consumption of cooking/table salt among the participants of the CESCAS cohort living in Bariloche and Marcos Paz (*n* = 3026). In addition, we administered a food frequency questionnaire to a random subsample of 2127 participants from these Argentinean cities. Data was collected from September 2013 to June 2014. 

### 2.2. Ethical Statement

All subjects gave their informed consent for inclusion before they participated in the study. The study was conducted in accordance with the Declaration of Helsinki, and the protocol was approved by the *Comité de Protocolos de Investigación del the Hospital Italiano de Buenos Aires* (Project 1489).

### 2.3. Dietary Assessment

Frequency of consumption and average portions of selected foods sources of sodium as well as their low-sodium alternatives were estimated by a short Food Frequency Questionnaire (FFQ) recently developed and validated in Argentina [21] for monitoring nutrition policies of reduction of sodium and trans fat recently implemented at the country. Items were selected based on categories of foods potentially affected by the new legislation, and local food composition and consumption data. The questionnaire was evaluated by expert nutritionists to assess face-validity, and then it was compared with three 24-h recalls. The short FFQ showed good inter-method reliability to estimate sodium intake from processed foods (paired *t*-test, *p* > 0.05; percent difference <10%; Spearman correlations > 0.5; cross-classification in opposite quartile < 10%), with no proportional bias (Bland–Altman correlation, *p* > 0.05) [21]. The short FFQ queries the frequency of intake of 34 food items during the last 12 months and asks the portion size for most of them (Table S1). Three food items represent low-sodium products that are currently available in the country (cheese, bread, and crackers without added salt), which are alternatives to frequently consumed foods with high sodium content. Average consumption of each food item per day was calculated based on frequency of consumption and portion size [22,23]. Sodium provided by each food item was calculated considering the average consumption of each food item per day and data of sodium content in food products [8,18,24,25] (Table S2).

Frequency of use of cooking/table salt was also assessed through two questions with 5 response options each (never, rarely, sometimes, often, and always).

The questionnaire was administered at home by trained interviewers. 

### 2.4. Covariables

Age, sex, and highest level of education data were collected. Marital status, health insurance coverage (yes/no), prevalence of hypertension (defined as systolic blood pressure ≥140 mm Hg and/or diastolic blood pressure ≥90 mm Hg, and/or use of antihypertensive medication), overweight and obesity (defined as body mass index >25 to ≤30 kg/m^2^ and >30 kg/m^2^), central obesity (defined as waist circumference ≥102 for men and ≥88 cm for women), smoking, and low physical activity (<600 MET-minutes/per week) were obtained from CESCAS I baseline database [19]. Blood pressure, height, weight, and waist circumference were measured by trained nurses at the baseline stage of the study.

### 2.5. Data Analysis

We estimated daily intake of sodium from selected foods overall and by subgroups of sex (male and female), age (35 to 54, 55 to 74 years old) and level of education (less than <8 years, 8–12 years, >12 years). We also estimated frequency of consumption, average intake/day, and average contribution of sodium from the selected food items and frequency of discretionary salt use. For descriptive purposes and to assess associations between discretionary salt use and demographic characteristics, response options were contracted into two categories: Never, rarely, or sometimes; and often or always. To assess associations, education level options were also contracted in two categories. Assessment of the association of demographic variables with sodium intake from processed foods was adjusted using multivariable linear regression. We derived odds ratios using multivariable logistic regression to assess associations of demographic variables with discretionary salt use. 

Data were analyzed using Stata/SE 12 (StataCorp, College Station, TX, USA) and all analyses were weighted considering the survey’s complex design.

## 3. Results

### 3.1. Characteristics of the Sample

Socio-demographic, lifestyle, and clinical characteristics of the participants are presented in Table 1.

### 3.2. Sodium Intake from Processed Foods

Table 2 shows the average daily sodium intake from the selected processed foods by sex, age, and level of education. The average intake of sodium from these foods was 1861 mg/day (4.7 g of salt), 1651 mg/day (4.1 g of salt) and 2098 mg/day (5.3 g of salt) overall, among women and men, respectively. Sex and level of education were independent predictors of sodium consumption (*p* < 0.001 and *p* = 0.006, Supplementary Table S3). Sodium intake from processed foods among men were 439 mg/day (95% CI: 304–574 mg/day) higher than women, and those with more than 8 years of formal education reported a mean consumption 192 mg/day (95% CI: 328–56 mg/day) lower.

### 3.3. Main Sources of Sodium Among Processed Food Groups and Food Categories Affected by the Current Legislation (National Act 26.905)

The frequency of consumption of each food item is described in Supplementary Table S4. Briefly, farinaceous such as breads and crackers were the food products more frequently consumed. Bread sold at bakeries was the product with the highest frequency of consumption. More than 70% of the participants reported they had consumed this type of bread at least once a week during the past 12 months and 26% had done so every day. Also, crackers were consumed by 21% of the population daily. Regarding alternatives with less sodium, almost 30% of participants reported consuming crackers without added salt at least once a week. Mean consumptions of without-added-salt crackers, breads, and cheeses were 6.8 (SE: 0.3), 8.3 (SE: 0.6), and 19 (SE: 0.8) g/day, and provided less than 1% of sodium from processed foods. Other processed foods such as cheeses and bouillon cubes/powders were consumed more than once a week by at least 25% of the population, and meat products such as cold cuts, and other convenient foods such as pizzas, empanadas/pies puff were consumed by near 45% of participants at least once a week. By contrast, less than 10% of the population reported consuming salted snacks every week.

**Figure 1:**
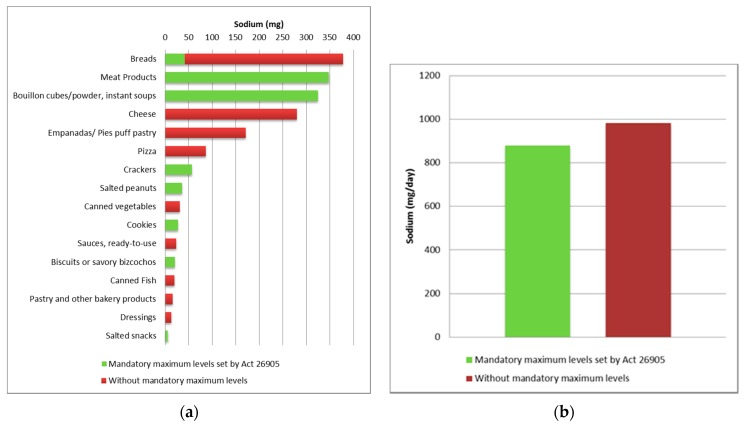
Dietary food categories sources of sodium and National Act 26.905: (**a**) Ranking of main dietary sources of sodium and food groups regulated by Act 26.905; (**b**) Sodium intakes from food categories with and without mandatory maximum levels.

**Figure 2:**
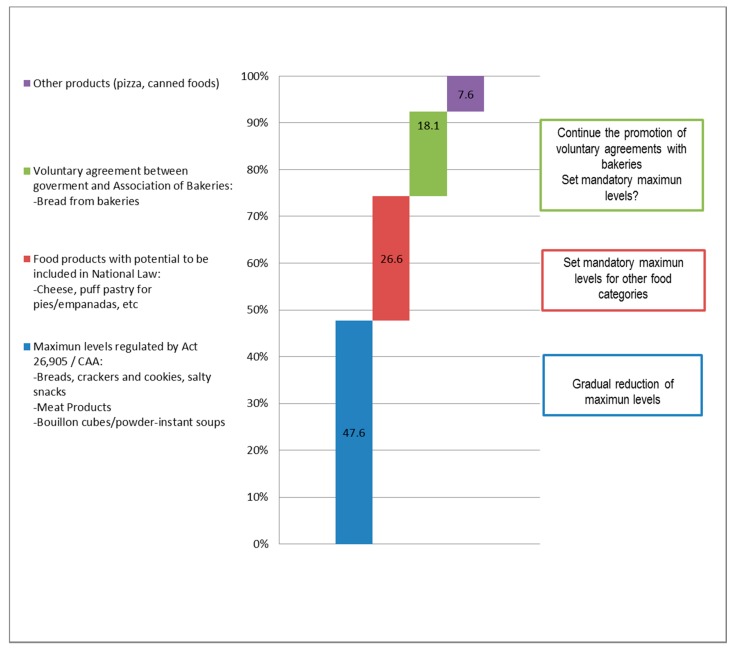
Main sources of sodium among food products and potential interventions to reduce the intakes at the population level.

Mean daily intakes and sodium provided from each product as well as their relative importance out of the total sodium from all processed foods in the questionnaire are summarized in Table 3 and Figure 1. In the same table, products were classified as regulated with maximum limits of sodium content by Act 26.905 or not regulated. The main food groups providing sodium were: “Soups and other convenience foods”, “Bread, crackers and cookies”, “Meat products”, and “Cheeses”. It was estimated that they accounted for 36.1%, 24.9%, 18.7%, and 15.0% of the sodium provided by processed foods, respectively. In particular, some food products were estimated to represent the major sources of sodium from processed foods in this population, including meat products (18.7%), bread from bakeries (18.1%), bouillon cubes/powder and instant soups (17.5%), cheeses (15%), and puff pastry for pies/empanadas (9.2%), as well as pizza (4.6%). Nearly half of the sodium (47.6%) was provided by products regulated by the law. Among those products not included in the law, French bread and other packaged foods products not yet regulated provided the rest of the salt. Figure 2 presents the main sources of sodium among food products and potential interventions to reduce the intakes at the population level.

Ranking of food sources among processed foods by age, sex, and educational level are presented in Supplementary Tables S5.1–S5.4. Bread, meat products, bouillon cubes/powder or instant soups, and chesses were the most important sources. In general, the first two food sources of sodium among men were bread from bakeries and meat products. Among women, bouillon cubes/powder or soups and meat products were the principal sources, but there were some differences of relative importance of food sources according to the educational attainment (e.g., bread intake was lower and cheese intake was higher at higher educational level). Food sources of sodium among women and low level of education were more frequently categories of foods with maximum limits included in the current national law. (Supplementary Material Figure S1).

### 3.4. Use of Discretionary Salt

The reported frequencies of use of salt at the table often or always, and in cooking were 9.9% and 73.3%, respectively (Table 4). In multivariable analysis (Supplementary Table S6), older adults were more likely to report avoiding salt use in cooking and at the table (OR of often/always adding salt in cooking: 0.66, 95% CI: 0.59–0.73; at the table: 0.67, 95% CI: 0.53–0.85). Men were more likely than women to report salt use at the table (OR: 1.38; 95% CI 1.09–1.73). There was no association of level of education with reported salt use in cooking or at the table. 

## 4. Discussion

This study identified sodium intake from selected food products and the top contributors to dietary sodium intake as well as frequency of discretional salt use in adults older than 35 years old in two Argentinean cities. Separate estimates for groups of age, sex, and level of education are also described. 

While bread from bakeries is known to be the major source of sodium in Argentina, our study set forth identifying other food sources of sodium to monitor and develop strategies to reduce sodium intake in our population. Once identified, we sought to study if these other sources had been included in the 2013 National Law. The food categories included in the law provided 2.1 g/day of salt (875 mg/day of sodium), with meat products and bouillon cubes/powders as top contributors (near 1.7 g/day of salt or 675 mg/day of sodium). As Allemandi et al. [18] have previously reported, based on sodium content declared in food labels, all the evaluated bouillon cubes and soups (*n* = 27) and almost all (51 out of 55) meat products were below the mandatory targets before the act was full implemented. Some snacks and packaged bakery products were also above the target in 2013–2014 [25]. In our study, if mean content of sodium in meat products, snacks, and packaged baked products that were above the target (Table S1) were reduced to mandatory maximum levels, the sodium intake would be reduced in less than 30 mg/day of sodium. In this scenario, food categories included in the National Law would provide 849 mg/day of sodium instead of 875 mg/day. Thus, reaching further reduction of sodium intake would require monitoring and also adjusting the maximum limits of these products.

In addition to the foods currently included in the law, we also identified other products that are contributors of sodium intake and could be incorporated into the regulation in the future. For instance, cheeses were important contributors to the overall sodium intake (0.7 g/day of salt or 280 mg of sodium), and therefore would be good candidates to be included in further regulations, mainly because there were previous experiences of voluntary agreement of progressive reduction of salt in these products [26]. Other food products categorized as convenience foods, such as puff pastry for “empanadas” (small filled pies) and pies/quiches, which are quite frequently consumed in Argentina, were moderate but consistent contributors of sodium (170 mg/day of sodium and 0.4 g/day of salt) and could be considered for salt reduction and setting of maximum levels of sodium content. 

As expected, artisanal bread, like French bread sold at bakeries, was frequently consumed and resulted an important contributor of sodium (336 mg/day sodium and 0.8 g/day of salt). These results were somewhat lower but still consistent with those estimated by the National Nutrition and Health Survey (ENNyS) conducted in 2004–2005 among women aged 10–49 [27] in Argentina, but represented nearly a fourth of the estimated national per capita consumption of 190 g/day in 2010 [28], based on the production and sales. Besides those differences, which could be explained by the different methodologies to measure salt intake such as food production/sales against food consumption, the national “Less Salt More Life” Program reported that between 2009 and 2015 more than 9000 traditional bakeries and hypermarkets had voluntarily adhered to 25% sodium reduction in breads [26]. We used in our work an estimation of 2% of salt in bread [8], however the current contribution of sodium may be lower since we are not considering the last Less Salt More Life actions. More efforts should be made to reach most of the bakeries at local levels. If the content of salt in bread from all bakeries was 1.5%, the reduction in sodium intake in this population would be of 84 mg/day or 0.21 g/day of salt. The baseline level of sodium in breads in Argentina was similar to other South American countries like Paraguay and Chile. Mandatory and voluntary reduction policies have been implemented in other Latin American countries. Initial reduction of salt in bread in those countries was similar to that implemented in Argentina (25%), but further reductions seem to be feasible, like the experience of Chile [29], and countries in other regions (e.g., UK, Ireland, and Australia) [30]. Most of the reduction of sodium in bread in Argentina was voluntary. If these reformulation efforts were mandatory in Argentina and maximum limits were the same as that for the packaged breads without bran (maximum limit of 501 mg/100 g), the reduction in sodium intake in this population would be of 126 mg/d or 0.31 g/d of salt. Based on previous estimations, both scenarios, as well as further reductions, would have a significant impact on the mortality and morbidity of the population [31].

In our work, discretionary salt use at the table proved to be in good agreement with results of the National Risk Factors Survey conducted in 2013 [7], including the gradient across age groups and gender. Moreover, the most remarkable result about discretionary salt use that emerged from our data was that almost 3/4 of the population reported they often/always use salt in cooking, with higher frequencies among younger adults. In our study, the percentage of people that have reported table salt usage often or very often was near the proportion reported in USA [32] and slightly lower than in Australia [33] and the UK [34]. However, adding salt “often or very often” during cooking was very much more frequent than reported in those countries. Nowson et al. [35] have reported a relationship between the discretionary salt used sometimes or often/always and higher salt excretion. Thus, implemented strategies to reduce the use of salt at the table should be maintained (e.g., education, interventions reducing salt shakers at the table in restaurants) though changing discretional use of salt in cooking may require other actions. The Argentinean Food Guidelines [36,37] explicitly recommend reducing salt use while cooking along with replacement with herbs and condiments. However, recent qualitative studies have reported that people trying to reduce the amount of salt may use strategies like using seasonings such as mayonnaise, balsamic vinegar, lemon, or consommés [17], some of them with high sodium-content such as bouillon cubes/powders, which were not recognized as sources of salt [16]. Additionally, other studies conducted in Argentina have reported that reducing consumption of salt tended to be a reactive event when personal/family health events took place, rather than a proactive behavior [16,38], and that people tended to perceive to have a high intake of salt based only on table salt [38]. 

In our study, sodium intake from processed foods almost reached the maximum recommended intake of salt, and also a high percentage of the population reported adding salt during cooking. While in developed regions, such as European and Northern American countries, sodium intake is dominated by sodium added in manufactured foods (≥75% of intake) [39], in Latin American countries both discretionary salt and processed foods can be identified as important sources of sodium [17]. For example, more than 70% of dietary sodium in Brazil is provided by salt and salt-based condiments added to foods [40]. However, consumption of processed foods and ready meals is rapidly growing [41] in Brazil and other countries of the region [42]. 

This study has several strengths. First, the sample is representative of the general population in those cities, and since it has been conducted immediately before the law entered into force, it can provide useful baseline data to allow monitoring of future progress. To our knowledge, this is the first study in our country to analyze sources of sodium in subgroups of adults of both sexes according to age and education level in a population-based sample. This information is very useful to adapt policies to facilitate and promote a healthy food environment to specific vulnerable subgroups of the population. Also, in this work, we have estimated sodium consumption provided by a list of foods from a short FFQ, which have shown good inter-method reliability to obtain population estimations compared with three 24-h recalls. There are also some limitations. Data about sodium content in food products were estimated mainly for indirect sources. However, this approach allows including information representative of sodium content of main available food products at one time point and could be then updated to perform comparisons. Also, these results need to be interpreted with caution because they might not be generalized to the national level or people younger than 35 years old. Regarding the questionnaire, reported sodium intake here represents only estimated sodium from processed foods, which is likely to be lower than the total sodium intake because of the exclusion of salt added in cooking, at the table, fresh foods, and from fresh foods, supplements, and medicines. Besides that, it has been the first application of this tool in a large-population study and the FFQ seems to be feasible to implement in other settings, or to repeat its application in the future in the same population to assess changes in consumption of processed food sources of sodium. Because the original questionnaire aims to assess sodium and trans fatty acids, some foods (e.g., margarine and butter) providing very low dietary sodium could be avoided in future applications. 

Identified food sources could be targets for interventions by reducing sodium content in these foods and/or reducing the intake of these products. Since salt in foods has many functions [43] and several actors are involved in reformulation processes, selection of new food categories to be included in the legislation and/or further reduction of sodium content in products already included should be made based on feasibility, opportunity, technology, and implementation issues [8,44,45]. It is a primary responsibility of the government to guide and facilitate these processes as it is its duty to provide relevant information and education that allow the population to make informed food choices, like the diffusion and updating of the national food guidelines and devising a more clear food labeling strategy [17,46].

## 5. Conclusions

In summary, the results of our study have helped to identify the main sources of sodium intake among adults in two Argentinean cities. The identification of these foods will be important for monitoring and developing strategies to reduce sodium intake in this population. It is concluded that in these Argentine cities, selected processed foods such as bread, meat products, bouillon cubes/powders, soups, cheeses, other convenience foods, and bakery products are important contributors of sodium to the adult’s diet in these Argentine cities, with some differences among sex and level of education. In addition, discretional salt use is very frequent, particularly in cooking among both sexes. Nearly half of the sodium from processed foods is estimated to have been provided by products with maximum limits set in 2013 by National Act 26.905. Based on our findings, government strategies to reduce salt consumption at the national population level are well directed, but further interventions are necessary to offer a healthy food environment and promote healthy food choices. Among them, the government should consider: (a) progressive reductions of already defined targets, which could positively influence sodium consumption of all the population, and particularly women and people with less educational level; (b) inclusion of maximum limits for other food products that represent important contributions of sodium such as cheese and bread rom bakeries; (c) development of new and clearer labeling strategies to allow the population to better identify food sources of salt as well as regulation of publicity of foods; (d) the launch of campaigns targeting salt use and other high-sodium ingredients in cooking such as bouillon cubes that are not recognized as food high in salt. Continuing the implementation of salt reduction policies in Argentina will contribute to the progress in preventing and controlling cardiovascular diseases and their associated risk factors and will also help accomplish global targets of reducing premature deaths from NCDs by 2025.

## Figures and Tables

**Table 1:** General characteristics of the study population.

Characteristics	Discretionary Salt Data (*n* = 3026) (%)	Short FFQ (*n* = 2129) (%)
Female	62.0	61.5
Age		
35–54 years	50.4	53.1
≥55 years	49.5	46.9
Education		
≤7 years	56.0	54.0
8–12 years	30.8	31.8
>12 years	13.2	14.2
Marital Status ^1^		
Married	67.7	68.6
Single, divorced or widowed	32.3	31.4
Health Insurance coverage	56.0	49.0
City		
Bariloche	46.2	42.8
Marcos Paz	53.8	57.2
Lifestyle and clinical characteristics ^1,2^		
Low physical activity (<600 MET-minutes/week)	22.7	23.4
Current smoker	27.2	28.4
Overweight (BMI ≥ 25 and < 30 kg/m^2^)	37.5	36.7
Obesity (BMI ≥ 30 kg/m^2^)	36.6	37.4
Central Obesity ^3^	49.6	48.5
Hypertension ^4^	43.9	41.4

BMI: Body mass index, FFQ: Food frequency questionnaire; ^1^ Data from baseline (2011–2012); ^2^ Weighted data; ^3^ Waist circumference ≥102 for men and ≥88 cm for women; ^4^ Systolic blood pressure ≥140 mm Hg and/or diastolic blood pressure ≥90 mm Hg and/or use of antihypertensive medication.

**Table 2:** Weighted mean, standard error (SE), and 95% confidence interval (95% CI) of estimates of daily intakes of sodium (mg) from food items included in the short FFQ by sex, age, and level of education.

	Overall (*n* = 2129)			Female (*n* = 1310)			Male (*n =* 819)		
	Mean (mg/day) ^1^	SE	(95% CI)	Mean (mg/day)	SE	(95% CI)	Mean (mg/day)	SE	(95% CI)
All	1861	33.7	(1795–1928)	1651	37.6	(1578–1725)	2098	56.9	(1987–2210)
Age									
<55 y	1875	45	(1788–1963)	1675	50	(1577–1772)	2104	76	(1956–2252)
≥55 y	1831	46	(1741–1921)	1604	52	(1503–1705)	2085	77	(1933–2237)
Education									
≤7 y	1966	48	(1871–2061)	1792	57	(1681–1903)	2147	79	(1991–2302)
8–12 y	1825	61	(1706–1944)	1575	64	(1449–1701)	2139	107	(1930–2349)
>12 y	1691	72	(1550–1831)	1492	77	(1341–1643)	1912	122	(1670–2151)

SE: Standard error; y: Years; ^1^ Values are expressed in mg of sodium; to calculate grams of salt, multiply mg of sodium by 0.0025.

**Table 3:** Mean intakes of food products and sodium by food categories with or without mandatory maximum limits regulated by National Act 26.905.

Food Groups	Food Intake/Person (g/Day or ml/Day) * Mean (SE)	Estimated Sodium Consumed per Person (mg/Day) Mean (SE)	Percentage of Sodium Provided by Food Category ^1^	Maximum Limit Set by Act 26.905
Soups, dressings, canned foods, and other convenience foods			36.1	
Bouillon cubes or powder	60 (1.9) *	213 (6.9)	11.5	Yes
Instant soups	47 (2.1) *	112 (5)	6.0	Yes
Puff pastry for pies/quiches	17 (0.5)	106 (3.3)	5.7	No
Puff pastry for *empanadas* ^2^	10.5 (0.3)	65 (1.6)	3.5	No
Pizza	17.1 (0.4)	86 (2.2)	4.6	No
Canned vegetables/legumes	15 (0.5)	32 (1.1)	1.7	No
Canned Fish	5.8 (0.2)	20 (0.6)	1.1	No
Sauces, ready-to-use	6.1 (0.2)	24 (0.8)	1.3	No
Dressings: Mayonnaise, mustard, ketchup, etc.	1.4 (0.1)	13 (0.6)	0.7	
Breads, crackers, cookies			24.9	
Bread, French or whole wheat (from bakeries)	42 (1.5)	336 (11.7)	18.1	No
Bread, Sliced or sandwich (packaged)	5.8 (0.4)	29 (1.8)	1.5	Yes
Bread, Vienna, hot dog/hamburger bun (packaged)	2.9 (0.2)	14 (1)	0.7	Yes
Crackers, white or whole wheat (packaged)	9.1 (0.4)	57 (2.5)	3.1	Yes
Cookies (packaged)	4.1 (0.2)	12 (0.6)	0.6	Yes
Filled/sandwich cookies such as Oreo	6.4 (0.3)	16 (0.8)	0.9	Yes
Meat Products			18.7	
Vienna sausage	5 (0.2)	49 (2.1)	2.6	Yes
Chorizo/Chorizo sausage/Argentinean sausage	8.5 (0.3)	89 (3.1)	4.8	Yes
Other cold cuts (such as ham, salami, bologna, etc.)	7.5 (0.2)	62 (2)	3.3	Yes
Pre-processed hamburgers	11.3 (0.5)	81 (3.6)	4.4	Yes
Frozen pre-cooked breaded chicken, nuggets or fish	12.8 (0.5)	66 (2.8)	3.5	Yes
Cheeses			15.0	
Soft cheeses	29.1 (1)	129 (4.4)	6.9	No
Semi-hard cheeses	21 (0.7)	131 (4.5)	7.0	No
Hard cheese	2.5 (0.1)	20 (0.5)	1.1	No
Pastry, other bakery products, sweets			2.0	
Biscuits or savory bizcochos	2.8 (0.1)	21 (0.9)	1.1	Yes ^3^
Cakes, pies, muffins, etc.	2.9 (0.1)	8 (0.4)	0.4	Yes
Danish/croissants and other Argentine facturas ^4^	9.2 (0.3)	5 (0.2)	0.3	No
Alfajores ^5^	2.9 (0.2)	3 (0.2)	0.2	No
Other			2.7	
Salted snacks	0.8 (0)	6 (0.3)	0.3	Yes
Salted peanuts	2.5 (0.1)	37 (1.6)	2.0	Yes
Butter/Margarine	2.1 (0.1)	3.8 (0.2)	0.2	No

SE: Standard error. * All values are g/day, except bouillon cubes and instant soup, which are expressed as ml/day of reconstituted product. ^1^ Percentage out of 1861 mg/day from the sum of these foods and alternative low-salt foods. ^2^ Small pie. ^3^ Partially, only packaged products. ^4^ Traditional pastry. ^5^ Sweet biscuit with filling.

**Table 4:** Weighted frequency, standard error (SE), and 95% confidence interval (95% CI) of discretional use of salt in cooking and at the table by sex, age, and level of education.

Characteristics	Overall (*n* = 3026)			Female (*n* = 1879)			Male (*n* = 1147)		
	%	SE	(95% CI)	%	SE	(95% CI)	%	SE	(95% CI)
Use of salt in cooking (often or always)									
All	73.3	0.9	(71.5–75.1)	72.7	1.1	(70.5–74.9)	74	1.5	(71.1–76.9)
Age									
≤55 y	79.4	1.2	(77.2–81.7)	78.6	1.4	(75.8–81.4)	80.3	1.9	(76.7–84)
>55 y	61.7	1.4	(59–64.4)	61.7	1.8	(58.2–65.2)	61.7	2.2	(57.5–66)
Education									
≤7 y	70.8	1.2	(68.3–73.2)	68.9	1.6	(65.8–72.0)	72.9	1.9	(69–76.7)
8–12 y	77.6	1.5	(74.6–80.6)	77.7	1.9	(74.0–81.03)	77.5	2.4	(72.7–82.3)
>12 y	71.5	2.5	(66.5–76.5)	72.5	3	(66.6–78.4)	70.3	4.2	(62.1–78.6)
Use of salt at the table (often or always)									
All	9.9	0.7	(8.5–11.3)	9.2	0.9	(7.5–10.9)	10.8	1.2	(8.5–13.1)
Age									
≤55 y	11.0	1.0	(9.0–12.9)	10.4	1.2	(8.1–12.8)	11.6	1.6	(8.4–14.7)
>55 y	8.0	0.8	(6.3–9.6)	6.8	1.0	(4.8–8.9)	9.3	1.4	(6.5–12.0)
Education									
≤7 y	8.6	0.9	(6.8–10.4)	8	1.1	(5.8–10.2)	9.3	1.5	(6.4–12.2)
8–12 y	10.5	1.2	(8.1–13)	10.1	1.6	(7–13.2)	11	2	(7.1–14.8)
>12 y	12.5	2	(8.5–16.5)	10.5	2.2	(6.1–14.9)	14.8	3.5	(7.9–21.6)

SE: Standard Error; y: years.

## Supplementary Materials

The following are available online at www.mdpi.com/2072-6643/9/9/964/s1, Table S1: Food items included in the short questionnaire, household measuring units to estimate quantities per time, sodium content per 100 g of food and maximum values set by National Act 26905; Table S2: Sodium content in food products/100 g of food: Source of data; Table S3: Weighted linear regression models of sodium intake from food products; Table S4: Reported weighted frequency of consumption of separated food items included in the short questionnaire during the last 12 months; Table S5.1: Main dietary sources of sodium among women by age group; Table S5.2: Main dietary sources of sodium among men by age group; Table S5.3: Main dietary sources of sodium among women by level of education; Table S5.4: Main dietary sources of sodium among men by level of education; Table S6: Multiple-adjusted odds ratios of adding salt associated with demographic characteristics in adults aged 35–74 years, Bariloche and Marcos Paz, Argentina, *n* = 3026; Figure S1: Sources of sodium from food products considering the inclusion in the National Act 26905, by sex and level of education.
