# Supplementary material for: Collecting Evidence to Inform Salt Reduction Policies in Argentina: Identifying Sources of Sodium Intake in Adults from a Population-Based Sample
Source: Nutrients. 2017 Aug 31;9(9):964. doi: 10.3390/nu9090964 (PMC5622724; doi:10.3390/nu9090964)
Supplement: Supplementary file 1 [file nutrients-09-00964-s001.pdf]

Supplementary Tables and Figures

Table S1. Food items included in the short questionnaire, household measuring units to estimate quantities per time, sodium content per 100 g of food and maximum values set by National Act 26905.

Food product	Units	Sodium (mg/100 g)	Max. values: Sodium mg/100 g
**Cheeses**			
Soft and semi-soft cheeses such as Brie or Port Salut	[slices, 1 slice=60g]	443	-
Medium-hard cheeses such as Gouda, Mar del Plata, Edam	[slices, 1 slice=60g]	624	-
Hard cheeses or "grating cheeses" such as Parmesan or Reggianito	SP (10 g)*	804	-
Cheese without added salt	SP (60 g)*	55.3	-
**Meats**			
Vienna sausage	[units, 1 unit=40 g]	978	1196
Chorizo or chorizo-sausage, fresh sausage	[units, 1 unit=90 g]	1041	950
Other cold cuts as ham salami bologna and other sliced cold cuts	[slices, 1 slice=20 g]	833	1196
Pre-processed hamburgers	[units, 1 unit=90 g]	720	850
Frozen pre-cooked breaded chicken nuggets or fish	[medallions, 1 medallion=82 g]	515	736
Canned fish	SP (60 g)*	346	-
**Dought**			
"Empanadas" (individual pies) , puff pastry	[units, 1 unit=30 g]	619	-
Quiches/pies (any filling), puff pastry	[servings, 1 serving=50 g]	625	-
Pizza	[servings, 1 serving=60 g]	505	-
**Breads, Crackers and Cookies**			
French bread or whole-wheat bread	["mignones"(bread rolls), 1 mignon=40 g]	800	-
Sliced or sandwich bread, white or whole-wheat	[slices, 1 slice=25 g]	494	530/501
Hamburger or hot dog buns	[units, 1 unit=50 g]	474	530
Bread without added salt	["mignones" (bread rolls), 1 mignon=40 g]	10	-
White or whole wheat crackers	[units, 1 unit=6 g]	629	941
Crackers without added salt made with white or whole flour	[units, 1 unit=6 g]		-
Cookies	[units, 1 unit=7 g]	283	512
Filled/Sandwich cookies such as Oreo®	[units, 1 unit=13 g]	251	429
Danish/croissants and other baked/fried sweet dough as churros	[units, 1 unit=35 g]	55	-
Biscuits or savory bizcochos	[units, 1 unit=11 g]	747	530
Cakes, pies, muffins, cupcakes	[servings or slices, 1 serving=40 g]	280	
Alfajor	[units, 1 unit=50 g]	100	-
**Other products**			
Margarines	[teaspoons, 1 teaspoon=5 g]	435	-
Butter, salted	[indiv. pack/teaspoons, 1 teaspoon=5 g]	160	-
Salted snacks (such as salted sticks, cheese puffs, chips)	[cups, 1 cup=25 g]	768	950
Salted peanuts	[cups, 1 cup=100 g]	1451	950
Sauces, ready-to-use (such as Italian or Spaghetti sauce)	SP (50 g)*	389	-
Canned vegetables and/or legumes (such as corn, garden salad, lentils)	SP (100 g)*	212	-
Bouillon cubes or powder	SP (quantity to prepare one serving of 200 ml)*	352	430
Instant soups	[individual pouches, cups or bowls, 1 cup =235 ml]	239	352
Mayonnaise, mustard, ketchup, etc	SP (12 g)*	916	-

*Quantities of these items were based on standardized portions (SP)

Table S2. Sodium content in food products/100g of food: Source of data

Food products	Source	Observations
French bread or whole-wheat bread , [bread rolls]	Ferrante et al 2011[2]	Direct Analysis<br>French bread.<br>Representative sample of bakeries from Argentina affiliated with the Argentinean Federation of Bakeries.
Cheeses, Meat Products (Vienna sausage, Chorizo or chorizo-sausage sausage, Other cold cuts as ham, salami, bologna and other sliced cold cuts, Pre-processed hamburgers, Frozen pre-cooked breaded chicken, nuggets). Canned fish. Canned vegetables. Bouillon cubes or powder, Instant soups. Empanadas/pies/quiches (puff pastry), Pizza Margarines, Butter (salted). Salted snacks, Salted peanuts. Sauces (ready-to-use). Alfajores. Cakes, pies, muffins, cupcakes.	Allemandi et al 2013[3]; Allemandi et al 2015[1]	Indirect Data (Labeling or food industry web site)<br>Sample based on market share (main brands were included) in one supermarket.
Sliced or sandwich bread, white or whole-wheat, Hamburger or hot dog buns, Bread without added salt, White or whole wheat crackers, Cookies, Filled/Sandwich cookies such as Oreo®, Crackers without added salt, made with white or whole flour, Biscuits or savory bizcochos	Elorriaga et al 2013[4]	Indirect Data (Labeling)<br>Packaged breads, crackers, cookies.<br>Any brand products available in 6 stores in Bariloche and Marcos Paz (2 supermarkets, two markets and two grocery stores).
Danish/croissants and other baked/fried sweet dough as churros	SARA Software[5]	Direct Analysis. (INFOODS Methodology)<br>Food codes A14, A15, A16

Table S3. Weighted linear regression models of sodium intake from food products.

	Sodium intake from food products(md/day)[1 2]	
	Coefficient (95%CI)	*P* value
Male	439.1 (304.8; 573.5)	<0.001
Age (≥55y)	-94.9 (-223.8; 334.0)	0.149
Education Level, >8y	-191.7 (-327.5; -55.8)	0.006

95% CI: 95% confidence interval.
[1]Values are expressed in mg of sodium per day; to calculate grams of salt, multiply mg of sodium by 0.0025
[2] Models included age, sex, level of education

Table S4. Reported weighted frequency of consumption of separated food items included in the short questionnaire during the last 12 months

Food items	Every day, once or more times (%)	2-6 times a week (%)	Once a week (%)	Less than once a week (%)	Never or less than once a month (%)
**Soups, dressings, canned foods and other convenience foods**					
Bouillon cubes or powder	8.9	21	16.3	19.9	33.9
Instant soups	9.1	7.0	9.6	20.9	53.4
Empanadas (meat pies)	0.1	13.9	32.4	46.2	7.4
Quiches (any filling such as vegetables, ham and cheese, poultry, etc)	1	20.0	24.7	36.8	17.5
Pizza	0.1	9.1	38.2	45.7	6.9
Canned vegetables/legumes	0.5	20.8	20.7	19.4	38.6
Canned Fish	0.0	7.9	22.7	39.9	29.5
Sauces, ready-to-use	0.2	18.5	18.7	15.5	47.1
Mayonnaise, mustard, kétchup, etc.	1.0	12.9	19.4	28.4	38.3
**Bread, crackers, cookies**					
French bread or whole wheat bread (from bakeries)	26.4	28.5	17.2	8.8	19.1
Sliced or sandwich bread	2.2	7.5	6.8	13.5	70
Bread, Vienna	0.3	2.4	5.7	9.6	82
Bread, any type without added salt	5.5	4.1	3.9	6.3	80.2
White or whole wheat crackers	21.3	11.8	8.1	14.6	44.2
White or whole wheat crackers, without added salt	16.6	7.3	5.4	12.2	58.5
Cookies	6	13.7	11.3	26.3	42.7
Filled/sandwich cookies such as Oreo	4.6	12.8	10.5	24.9	47.2
**Meats Products**					
Vienna sausage	0.0	6.3	13.7	30.8	49.2
Chorizo/Chorizo sausage /Argentinean sausage	0.1	1.7	19.8	48.2	30.2
Other cold cuts (such as ham, salami, bologna, etc.)	0.9	22	19.7	38.3	19.1
Pre-processed hamburgers	0.0	8.2	13.4	25.4	53
Frozen pre-cooked breaded chicken, nuggets or fish	0.2	11.7	12.8	12.8	62.5
**Cheeses**					

Soft cheeses (not including pizza, pies and cakes)	4.8	29.9	23.8	29.2	12.3
Semi-hard cheeses	1.7	23.8	22	32.3	20.2
Hard cheese (grated cheese type), alone or as part of another dish *	1.6	30.2	23.9	30.1	14.2
Without added salt	16.1	6.5	5.4	13.7	58.3
**Pastry, other bakery products, sweets**					
Danish/croissants and other "Argentine facturas"	1.7	12.2	22.4	37.9	25.8
Biscuits or savory bizcochos	3.7	11.1	9.1	19.4	56.7
Cakes, pies, muffins, etc	0.3	1.7	9	41.1	47.9
Alfajores	1.4	3.5	5.9	15.2	74
**Other**					
Margarine	1.0	2.2	3.8	6	87
Butter	4.9	20.8	18.3	10.5	45.5
Salted snacks	0.0	1	7.2	24.8	67.0
Salted peanuts	0.0	0.7	6	24.2	69.1

Table S5.1 Main dietary sources of sodium among women by age group

Food products	Age					
	< 55 y (n=724)			≥55 years (n=586)		
	Sodium consumed per person mg/day Mean (SE)	%	Ranking	Sodium consumed per person mg/day Mean (SE)	%	Ranking
Sodium from food products	1675 (50)		0	1604 (52)		
Bouillon cubes, powder/ Instant soups	**322 (16)**	**19.2**	**1**	**363 (19)**	**22.6**	**1**
Meat products	310 (14)	18.5	2	249 (12)	15.6	2
Traditional Bread (from bakery)	268 (15)	16	3	236 (18)	14.7	4
Cheeses	252 (11)	15.1	4	247 (12)	15.4	3
Empanada/Pie, puff pastry	148 (6)	8.8	5	153 (7)	9.5	5
Pizza	74 (3)	4.4	6	69 (3)	4.3	6
Crackers	57 (4)	3.4	7	57 (4)	3.5	7
Canned foods, (vegetables/fish)	49 (2)	2.9	8	56 (4)	3.5	8
Peanuts, salty	33 (2)	2	9	26 (3)	1.6	10
Sliced or sandwich bread	27 (3)	1.6	10	29 (3)	1.8	9
Cookies	26 (2)	1.5	11	18 (1)	1.1	12
Sauces, ready- to-use	24 (1)	1.4	12	22 (1)	1.4	11
Biscuits or savory bizcochos	18 (1)	1.1	13	16 (1)	1	13
Pastry and other	16 (1)	0.9	14	13 (1)	0.8	14
Seasonings	12 (1)	0.7	15	8 (1)	0.5	16
Bread, (Vienna, Hamb./ hot dog bun)	12 (1)	0.7	16	13 (2)	0.8	15

SE: Standard Error

Table S5.2 Main dietary sources of sodium among men by age group

Food products	Age					
	< 55 y (n=406)			≥55 years (n=413)		
	Sodium consumed per person mg/day Mean (SE)	%	Ranking	Sodium consumed per person mg/day Mean (SE)	%	Ranking
Sodium from food products	2104 (76)		0	2085 (77)		
Meat products	**427 (22)**	**20.3**	**1**	378 (20)	18.1	2
Traditional Bread (from bakery)	425 (27)	20.2	2	**423 (29)**	**20.3**	**1**
Cheeses	318 (18)	15.1	3	301 (17)	14.5	4
Bouillon cubes, powder/ Instant soups	290 (21)	13.8	4	367 (22)	17.6	3
Empanada/Pie, puff pastry	197 (10)	9.3	5	194 (10)	9.3	5
Pizza	105 (5)	5	6	94 (5)	4.5	6
Crackers	55 (5)	2.6	7	60 (5)	2.9	7
Canned foods, (vegetables/fish)	51 (3)	2.4	8	56 (3)	2.7	8
Peanuts, salty	47 (4)	2.2	9	35 (3)	1.7	9
Cookies	35 (3)	1.6	10	27 (3)	1.3	11
Sliced or sandwich bread	29 (4)	1.4	11	32 (4)	1.6	10
Biscuits or savory bizcochos	26 (2)	1.3	12	20 (2)	1	13
Sauces, ready- to-use	24 (2)	1.1	13	26 (2)	1.2	12
Pastry and other	18 (1)	0.9	14	16 (1)	0.8	14
Seasonings	17 (1)	0.8	15	12 (1)	0.6	16
Bread, (Vienna, Hamb./ hot dog bun)	15 (2)	0.7	16	16 (3)	0.8	15

SE: Standard Error

Table S5.3 Main dietary sources of sodium among women by level of education

Food products	Education								
	< 8years (n=684)			8-12 years (n 434)			>12 years (n 192)		
	**Sodium consumed per person mg/day Mean (SE)**	**%**	**Ranking**	**Sodium consumed per person mg/day Mean (SE)**	**%**	**Ranking**	**Sodium consumed per person mg/day Mean (SE)**	**%**	**Ranking**
Sodium from food products	1792 (57)		-	1578 (64)		-	1492 (77)		-
Bouillon cubes, powder/ Instant soups	**397 (22)**	**22.2**	**1**	285 (18)	18.1	2	296 (29)	19.8	2
Meat products	311 (13)	17.3	2	**304 (19)**	**19.3**	**1**	220 (21)	14.8	3
Traditional Bread (from bakery)	296 (21)	16.5	3	243 (18)	15.4	4	203 (22)	13.6	4
Chesses	235 (11)	13.1	4	245 (15)	15.5	3	**299 (25)**	**20.0**	**1**
Empanada/Pie, puff pastry	161 (7)	9	5	146 (8)	9.3	5	132 (12)	8.9	5
Pizza	74 (3)	4.1	6	74 (4)	4.7	6	68 (4)	4.5	6
Crackers	61 (5)	3.4	7	54 (5)	3.4	7	54 (7)	3.6	7
Canned foods, (vegetables/fish)	56 (2)	3.1	8	47 (2)	3	8	50 (7)	3.3	8
Peanuts, salty	31 (3)	1.7	9	34 (3)	2.1	9	25 (4)	1.7	10
Sliced or sandwich bread	29 (3)	1.6	10	22 (3)	1.4	11	35 (8)	2.4	9
Cookies	27 (2)	1.5	11	21 (2)	1.3	12	19 (4)	1.3	11
Sauces, ready- to-use	26 (2)	1.5	12	23 (2)	1.5	10	17 (2)	1.1	12
Biscuits or savory bizcochos	20 (2)	1.1	13	15 (1)	1.0	14	15 (3)	1.0	14
Bread, (Vienna, Hamburguer or hot dog bun)	15 (2)	0.8	14	12 (2)	0.8	15	8 (2)	0.5	16
Pastry and other	14 (1)	0.8	15	16 (1)	1.0	13	13 (1)	0.9	15
Seasonings	9 (1)	0.8	16	11 (1)	0.6	16	15 (2)	0.7	13

SE: Standard Error

Table S5.4 Main dietary sources of sodium among men by level of education

Food products	Education								
	**< 8years (*n* 465)**			**8-12 years (*n* 243)**			**>12 years (*n* 111)**		
	**Sodium consumed per person mg/day Mean (SE)**	**%**	**Ranking**	**Sodium consumed per person mg/day Mean (SE)**	**%**	**Ranking**	**Sodium consumed per person mg/day Mean (SE)**	**%**	**Ranking**
Sodium from food products	2147 (79)		-	2138 (107)			1912 (123)		
Traditional Bread (from bakery)	**426 (26)**	**19.9**	**1**	**450 (39)**	**21**	1	**375 (51)**	**19.6**	1
Meat products	414 (21)	19.3	2	439 (32)	20.6	2	357 (37)	18.7	2
Bouillon cubes, powder/ Instant soups	385 (27)	17.9	3	260 (22)	12.2	4	247 (28)	12.9	4
Cheeses	282 (15)	13.1	4	346 (28)	16.2	3	326 (30)	17.1	3
Empanada/Pie, puff pastry	201 (11)	9.3	5	196 (13)	9.1	5	184 (17)	9.6	5
Pizza	97 (5)	4.5	6	104 (8)	4.9	6	109 (9)	5.7	6
Canned foods, (vegetables/fish)	59 (3)	2.7	7	48 (3)	2.2	8	48 (5)	2.5	8
Crackers	57 (5)	2.6	8	56 (7)	2.6	7	58 (10)	3	7
Peanuts, salty	42 (3)	1.9	9	44 (5)	2.1	9	43 (7)	2.3	9
Cookies	32 (3)	1.5	10	33 (4)	1.5	10	32 (6)	1.7	10
Sliced or sandwich bread	29 (4)	1.4	11	31 (5)	1.5	11	30 (8)	1.6	11
Sauces, ready- to-use	27 (2)	1.3	12	24 (2)	1.1	13	18 (3)	1	13
Biscuits or savory bizcochos	25 (2)	1.2	13	28 (4)	1.3	12	17 (3)	0.9	14
Bread, (Vienna, Hamb./hot dog bun)	17 (3)	0.8	14	15 (3)	0.7	16	9 (2)	0.5	16
Pastry and other	15 (1)	0.7	15	21 (2)	1	14	16 (2)	0.8	15
Seasonings	12 (1)	0.5	16	18 (2)	0.8	15	22 (3)	1.1	12

SE: Standard Error

Figure S1. Sources of sodium from food products considering the inclusion in the National Act 26905, by sex and level of education

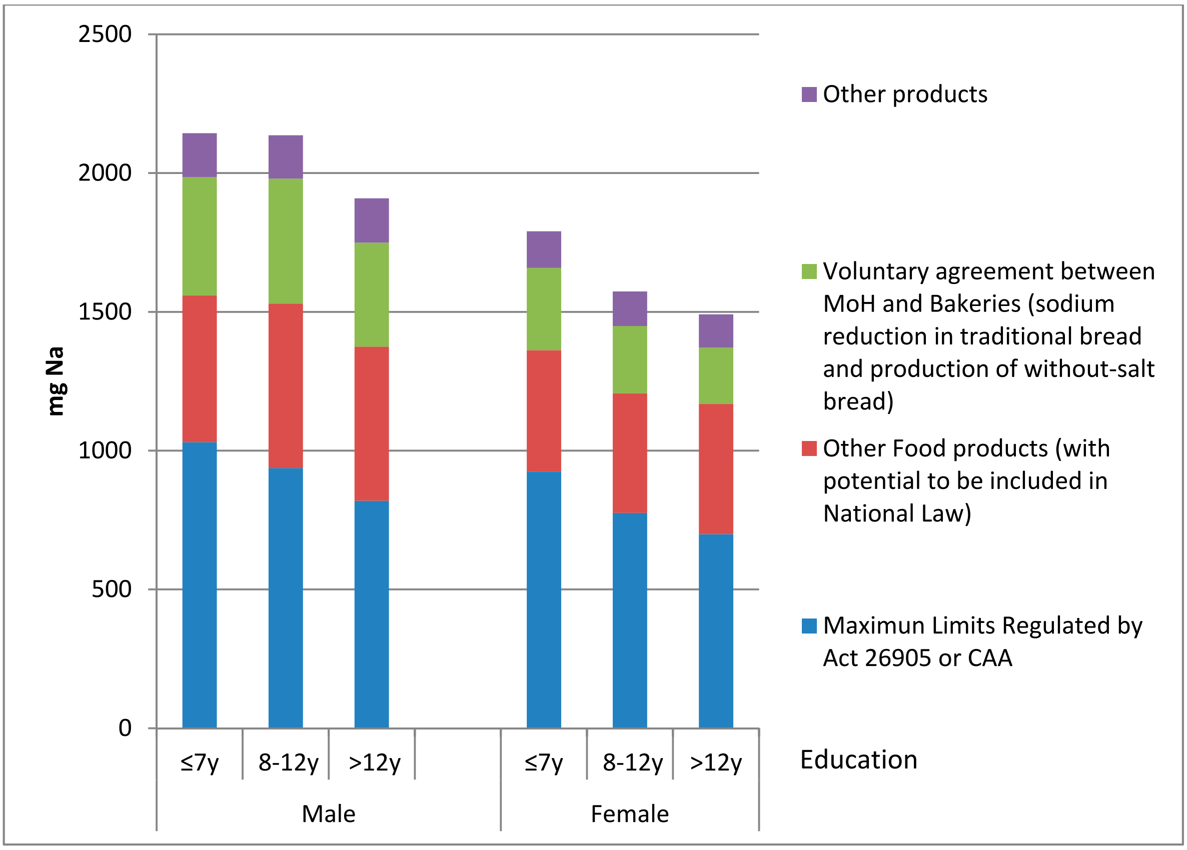

Table S6. Multiple-adjusted odds ratios of adding salt associated with demographic characteristics in adults aged 35-74y, Bariloche and Marcos Paz, Argentina, n=3026.[a]

	**Adding salt in cooking**[b]			**Adding salt at the table**[b]		
	**OR**	**(95%CI)**	***P* value**	**OR**	**(95%CI)**	***P* value**
**Male**	0.96	(0.78 – 1.20)	0.735	1.38	(1.09 -1.73)	0.007
**Age** (>55y)	0.66	(0.59 - 0.73)	<0.001	0.67	(0.53 – 0.85)	0.001
**Education Level**						
≥8 y	0.90	(0.72 – 1.13)	0.363	0.99	(0.78 – 1.26)	0.940

OR: Odds Ratio; (95%CI): 95% Confidence Interval
[a] Model included age, sex, level of education and city.
[b] Often /always.

References

1. Allemandi, L.; Tiscornia, M.V.; Ponce, M.; Castronuovo, L.; Dunford, E.; Schoj, V. Sodium content in processed foods in Argentina: Compliance with the national law. *Cardiovasc Diagn Ther* **2015**, *5*, 197-206.
2. Ferrante, D.; Apro, N.; Ferreira, V.; Virgolini, M.; Aguilar, V.; Sosa, M.; Perel, P.; Casas, J. Feasibility of salt reduction in processed foods in Argentina. *Revista panamericana de salud publica = Pan American journal of public health* **2011**, *29*, 69-75.
3. Allemandi, L.; Garipe, L.; Schoj, V.; Pizarro, M.; Tambussi, A. Análisis del contenido de sodio y grasas trans de los alimentos industrializados en Argentina. [analysis of sodium and trans fat content of industrialized foods in Argentina]. In *Rev Argent Salud Pública*, Buenos Aires, 2013; Vol. 4, pp 14-19.
4. Elorriaga, N.; Bardach, A.E.; Defago, M.D.; Levi, L.; Nessier, M.C.; Irazola, V. Development, implementation and evaluation of tools for decision-making on healthy eating policies in Argentina. In *Anuario Becas de Investigación "Carrillo Oñativia" 2012*, Ministerio de Salud: Buenos Aires, 2016; pp 250-251.
5. Ministerio de Salud de la Nación. S.A.R.A. Sistema de análisis y registro de alimentos. Programa informático para análisis de encuestas alimentarias – sara. http://msal.gov.ar/htm/Site/ennys/site/sara.asp
